# Mechanism and application of *Akkermansia muciniphila* in inflammatory diseases

**DOI:** 10.3389/fcimb.2026.1819005

**Published:** 2026-05-22

**Authors:** Luyu Xie, Juane Lu, Huan Han, Zengli Liu, Chang Dong, Hao Wu, Jianjun Qiao

**Affiliations:** 1 School of Synthetic Biology and Biomanufacturing, Tianjin University, Tianjin, China; 2 Zhe Jiang Institute of Tianjin University, Shaoxing, China; 3 State Key Laboratory of Synthetic Biology, Tianjin University, Tianjin, China

**Keywords:** Akkermansia muciniphila, application, chronic inflammatory diseases, mechanism, next-generation probiotic

## Abstract

Chronic inflammatory diseases, closely linked to gut microbiota dysbiosis, pose a major global health burden. *Akkermansia muciniphila* (*A. muciniphila*), a key gut commensal, sustains immune homeostasis and metabolic balance, emerging as a promising next-generation probiotic. This review synthesizes current evidence on the mechanisms such as regulating the intestinal barrier, immunomodulation, mediating anti-inflammatory metabolites and regulating microbiota and therapeutic potential of *A. muciniphila* in inflammation-related diseases. Integrating these with experimental/clinical data across inflammation-related disorders establishes a coherent framework. Beyond live bacteria, advances in pasteurized formulations and bioactive derivatives highlight complementary advantages.

## 1 Introduction

Inflammation-related diseases encompass a range of pathological conditions driven by immune responses to either pathogens or to the body’s own tissues, representing a significant global health challenge. According to the Global Burden of Disease study, the incidence of such disorders has increased in recent decades, particularly in developed nations (Park et al., 2023; Wang et al., 2023b). Based on the role of inflammation in disease onset, these conditions can be broadly classified into primary inflammatory diseases and inflammation-induced disorders.

Primary inflammatory diseases are characterized by inflammation that directly triggers tissue damage and pathological alterations, as seen in inflammatory bowel disease (IBD), chronic pancreatitis, and non-alcoholic steatohepatitis (NASH). In contrast, inflammation-induced disorders involve persistent, low-grade systemic immune activation that promotes disease progression. Representative conditions include type 2 diabetes (T2DM), Alzheimer’s disease, Parkinson’s disease (PD), and depression.

In recent years, extensive research has identified that the gut microbiota as a central regulator of inflammatory processes. Microbial dysbiosis can disrupt the mucus barrier and the expression of tight junction proteins, thereby increasing intestinal permeability. This compromise allows bacterial components such as lipopolysaccharide (LPS) to enter the bloodstream and activate immune pathways, resulting in systemic inflammation (Lupp et al., 2007). In patients with IBD, a marked reduction in short-chain fatty acid (SCFA)–producing taxa and mucus-degrading microorganisms is commonly observed (Zheng et al., 2025). Modulating microbial composition, for instance through fecal microbiota transplantation, has been shown to markedly attenuate colitis-associated inflammation in experimental models (Wen et al., 2023). Moreover, SCFAs generated by intestinal microbes enhance regulatory T-cell (Treg) activity and suppress chronic inflammatory signaling (Smith et al., 2013). Collectively, these findings indicate that preserving gut microbial balance is essential not only for controlling primary inflammatory disorders but also for slowing the progression of inflammation-associated conditions.

Probiotics, live microorganisms that help maintain intestinal balance and support metabolic health, have attracted growing scientific interest. Among them, *A. muciniphila* stands out for its distinctive metabolic and immunological features. Unlike conventional strains that depend on fermentable carbohydrates, it derives energy from mucin degradation, thereby contributing directly to mucosal maintenance. In addition, *A. muciniphila* exhibits unique immunoregulatory capabilities: its membrane phospholipids interact with host cells to reinforce barrier integrity and fine-tune immune signaling (Bae et al., 2022). It also possesses potent anti-inflammatory properties, mitigating pathological immune activation by modulating cytokine profiles. These attributes collectively position *A. muciniphila* as a promising next-generation probiotic with considerable potential in the prevention and management of chronic inflammation–related conditions.

While previous reviews have mainly focused on the association between *A. muciniphila* and individual diseases, a comprehensive synthesis integrating mechanistic insights, multi-omics evidence, microbial diversity within the *Akkermansia* genus, and emerging therapeutic strategies is still lacking. In this review, we summarize recent advances in understanding the anti-inflammatory mechanisms of *A. muciniphila*. By integrating mechanistic insights with translational advances, this work seeks to expand current understanding and provide a comprehensive framework for evaluating the organism’s therapeutic promise as a next-generation probiotic.

## 2 Overview of *A. muciniphila*

**Figure 1:**
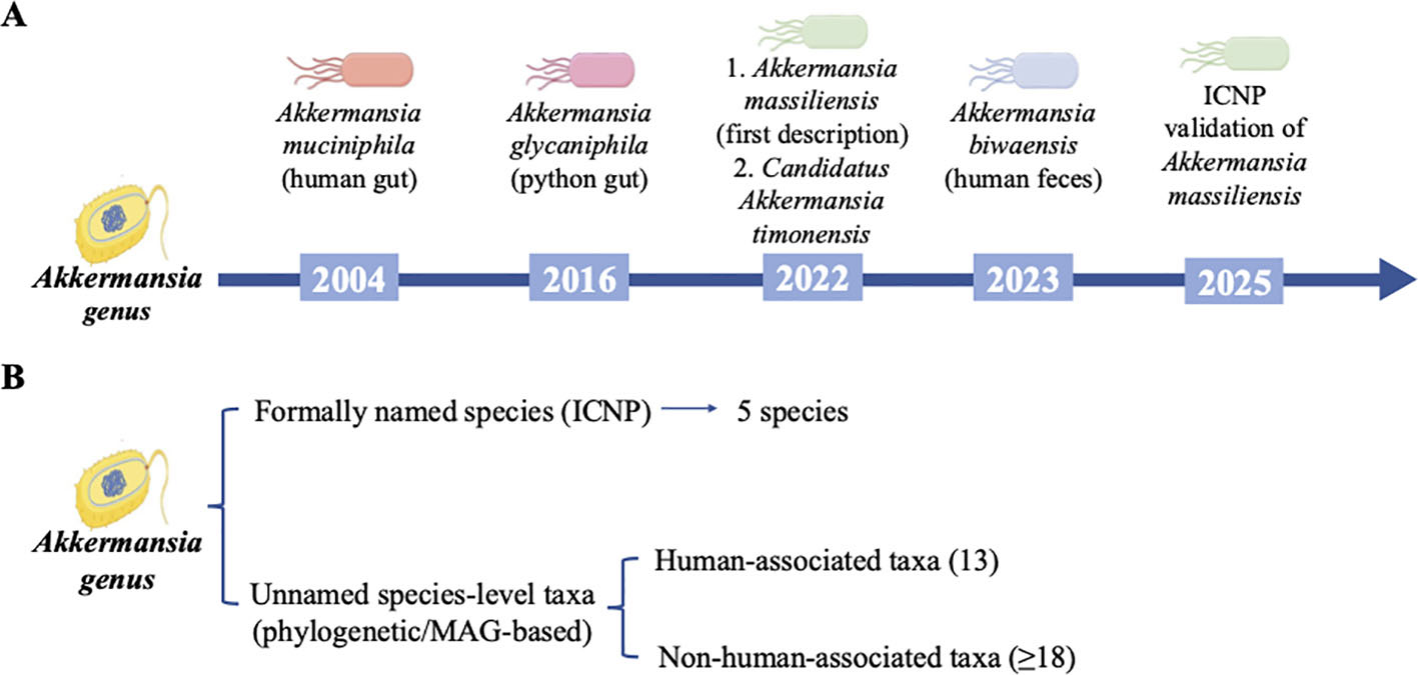
Taxonomic overview of the genus *Akkermansia*. **(A)** Timeline of *Akkermansia* species that have been formally described and validly published according to the International Code of Nomenclature of Prokaryotes (ICNP). To date, five species have been recognized, including *Akkermansia muciniphila*, *Akkermansia glycaniphila*, *Akkermansia massiliensis*, *Candidatus Akkermansia timonensis* and *Akkermansia biwaensis*. **(B)** Overview of the phylogenetic and metagenomic diversity of the *Akkermansia* genus. Recent metagenomic and phylogenetic analyses have identified at least 31 phylogenetically distinct species-level taxa, most of which remain unnamed. Among these taxa, 13 are consistently detected in the human gut.

According to the International Code of Nomenclature of Prokaryotes, five species within the genus *Akkermansia* have been formally recognized to date: *Akkermansia muciniphila*, *Akkermansia glycaniphila*, *Akkermansia biwaensis*, *Akkermansia massiliensis* and *Candidatus Akkermansia timonensis* (**Figure 1A**). *Akkermansia glycaniphila* was first isolated from the intestinal tract of a python (Ouwerkerk et al., 2016), whereas *Akkermansia biwaensis* was originated from the fecal samples of healthy Japanese adults (Kobayashi et al., 2023). *Akkermansia massiliensis* sp. *nov.* obtained from human intestinal specimens, possesses the unique ability to synthesize vitamin B_12_ autonomously, a feature distinguishing it from *A. muciniphila*. Its type strain is Marseiler-P6666T (= CSUR P6666 = CECT 30548) (Ndongo et al., 2022). However, recent metagenomic and phylogenetic analyses have revealed considerable genetic heterogeneity within the genus *Akkermansia* across multiple hosts, identifying at least 31 phylogenetically distinct species, 13 of which are consistently detected in the human intestine (**Figure 1B**) (Molteni et al., 2025). These findings suggest that the true diversity of this genus has likely been substantially underestimated and underscore the need for further research into species-specific contributions to host inflammation and metabolic regulation.

Among the recognized members of this genus, *A. muciniphila* is the most extensively investigated. It is a widely distributed microorganism belonging to the phylum *Verrucomicrobiota*, class *Verrucomicrobia*, order *Verrucomicrobiales* and family *Akkermansiaceae*. It was first isolated and identified from human feces by Derrien et al. in 2004 (Derrien et al., 2004). This Gram-negative anaerobe has an oval morphology, measuring approximately 0.5-1 μm and predominantly inhabits the mucosal layer of the human and other mammalian intestines. *A. muciniphila* utilizes mucin as its primary carbon and nitrogen source, and the degradation products generated during this process serve as metabolic substrates for neighboring commensal taxa. Through this ecological interaction, *A. muciniphila* contributes significantly to maintaining the intestinal barrier and regulating host immune activity.

Genomic analyses have identified a wide array of genes associated with mucin degradation (Van Passel et al., 2011), including those encoding proteases, sialidases and glycoside hydrolases, enabling the bacterium to influence the intestinal microenvironment. A growing body of preclinical and clinical evidence demonstrates a strong association between *A. muciniphila* abundance and intestinal mucus layer thickness, epithelial barrier function, and immune regulation. These findings highlight its pivotal role in the pathogenesis and progression of inflammation-related disorders and provide a foundation for elucidating its underlying mechanisms in immunological regulation.

## 3 The mechanism of *A. muciniphila* in inflammatory diseases

The beneficial effects of *A. muciniphila* are mediated through multiple interconnected mechanisms, including maintain the intestinal barrier function, immunoregulatory effect, metabolite-mediated anti-inflammatory effects, and regulation of gut microbial communities. Importantly, these mechanisms do not operate independently but rather interact with each other to form an integrated regulatory network that collectively influences host metabolic and inflammatory homeostasis.

These interconnected mechanisms are discussed in detail below.

### 3.1 Maintain the intestinal barrier function

Studies have shown that *A. muciniphila* fortifies the physical barrier by stimulating mucus secretion and increasing goblet cell numbers, thereby limiting the translocation of pathogens and other harmful substances (Sparfel et al., 2024; Zheng et al., 2023a). Its colonization on the epithelial surface improves cellular resistance, promotes epithelial proliferation, and facilitating mucosal repair while preserving tight junction protein structure (Reunanen et al., 2015). In addition, its metabolic by-products, particularly SCFAs, contribute to epithelial renewal, support intestinal barrier function, increase mucus layer thickness and serve as energy substrates for commensal bacteria (Robertson et al., 2005). SCFAs interact with GPCRs such as GPR41 and GPR43 on intestinal epithelial cells, initiating downstream signaling cascades. Activation of the cAMP-PKA pathway via GPR43, for instance, upregulates tight junction proteins, decreases epithelial permeability, and strengthens the overall gut barrier (Kim et al., 2013; Dalile et al., 2019; Shin et al., 2023). Recent research indicates that *A. muciniphila* contributes to intestinal barrier restoration by regulating intestinal stem cell (ISC) activity. Its mucin-degrading enzymes modulate mucus layer thickness, enhancing the accessibility of stem cells to essential nutrients and thereby promoting their proliferation and differentiation (Shuoker et al., 2023). The secreted protein Amuc_1409 interacts with E-cadherin to facilitate the dissociation of the E-cadherin/β-catenin complex, activating the Wnt/β-catenin signaling pathway and enhancing ISC-mediated intestinal regeneration (Kang et al., 2024). In addition, SCFAs and other metabolites function as signaling molecules that influence stem cell energy metabolism and epigenetic modifications, further reinforcing the regenerative capacity of the intestinal mucosa (Ma et al., 2025). Beyond live cells, pasteurized *A. muciniphila* and its derived components also demonstrate significant barrier-protective properties. Studies have shown that these elements mitigate LPS-induced increase in epithelial permeability and inflammation through TLR2-mediated AMPK and NF-κB signaling pathways, thereby improving intestinal barrier dysfunction (Wang et al., 2021; Shi et al., 2022). Moreover, bile acid metabolites such as 3-succinoyl cholic acid (3-sucCA) have been found to promote *A. muciniphila* colonization, indirectly strengthening mucosal integrity and attenuating systemic low-grade inflammation, which provides protection against metabolic-associated steatohepatitis (MASH) (Nie et al., 2024). Collectively, these findings highlight the bacterium’s pivotal role in restoring intestinal homeostasis and facilitating epithelial and stem cell regeneration through reinforcement of the mucosal barrier.

Improvement of intestinal barrier integrity may subsequently influence host immune responses by reducing microbial translocation and endotoxin exposure, thereby contributing to immune homeostasis.

### 3.2 Immunoregulatory effect

**Figure 2:**
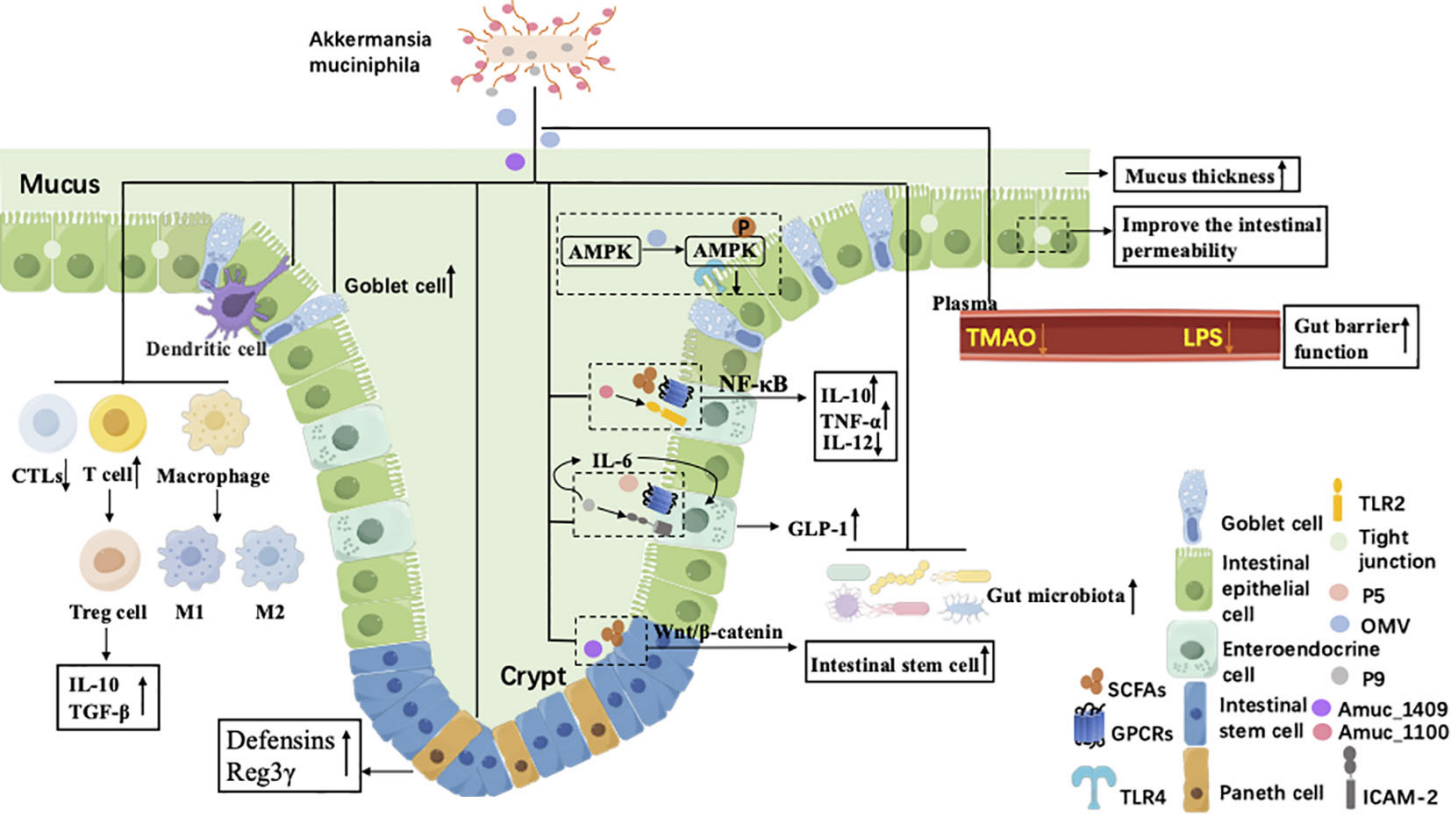
Brief overview of the anti-inflammatory mechanism of *A. muciniphila*. *A. muciniphila* exerts anti-inflammatory effects through coordinated and interconnected mechanisms, including enhancement of intestinal mucus secretion and tight junction protein expression to reinforce intestinal barrier integrity; modulation of the host immune system via interactions with immune cell receptors, leading to promoted Treg differentiation and suppressed pro-inflammatory cytokine production; regulation of local and systemic inflammation through microbial metabolites and host metabolic pathways; and remodeling of gut microbiota composition to maintain microbial homeostasis, collectively contributing to inflammation resolution and restoration of intestinal homeostasis.

The immunomodulatory effect of *A. muciniphila* primarily arises from direct interaction between its structural constituents, such as outer membrane proteins, secreted protein, lipooligosaccharide (LOS) and outer membrane vesicles (OMVs), and host immune cell receptor. These interactions activate specific signaling cascades, promote the proliferation and activation of Treg and the polarization of macrophages, and modulate cytokine secretion dynamics, ultimately producing either pro- or anti-inflammatory outcomes (**Figure 2**).

#### 3.2.1 The outer membrane protein

The outer membrane protein Amuc_1100 of *A. muciniphila* activates the NF-κB signaling cascade through Toll-like receptor 2 (TLR2), thereby modulating immune cell function and inflammatory cytokines secretion (Ottman et al., 2017). The interaction between Amuc_1100 and TLR2 has been shown to regulate obesity and improve insulin resistance in diabetic mouse models (Plovier et al., 2017; Song et al., 2023). Amuc_1100 can also prevent the progression of NASH by regulating TLR2 to activate γδT17 cells and the polarization of macrophages (Han et al., 2023). Additionally, Amuc_1100 can attenuate colonic inflammation by activating the aryl hydrocarbon receptor (AhR) pathway through modulation of tryptophan metabolism, and by regulating CD8^+^ cytotoxic T cells (CTLs), thereby alleviating colitis and preventing colitis-associated colorectal tumorigenesis (Wang et al., 2020; Gu et al., 2021). Beyond Amuc_1100, other membrane-associated proteins exhibit distinct bioactivities. Amuc_1434 inhibits LS174T cell proliferation through TRAIL-mediated apoptosis and degrades Muc2 mucin, contributing to colon cancer control (Meng et al., 2020). Amuc_2172 enters colorectal cells via micropinocytosis, induces heat shock protein 70 expression, and enhances CTL-mediated immune responses to suppress colorectal cancer (Jiang et al., 2023).

#### 3.2.2 The secreted protein

Several secreted proteins also display significant regulatory potential. The protein P9 binds intercellular adhesion molecule-2, stimulating glucagon-like peptide-1 (GLP-1) release and promoting interleukin-6 (IL-6) production, thus displaying anti-inflammatory properties (Payahoo et al., 2019; Yoon et al., 2021). P9 also promotes GLP-1 secretion and brown adipose tissue thermogenesis, contributing to improved glycemic regulation and obesity prevention (Yoon et al., 2021). Similarly, the P5 protein derived from pasteurized *A. muciniphila* enhances GLP-1 synthesis via G-protein-coupled receptor (GPCR) activation, thereby ameliorating T2DM symptoms (Niu et al., 2024). Moreover, Kim et al. identified the secreted protein AmTARS, which specifically binds to TLR2, triggering MAPK and PI3K/AKT signaling pathways that promote CREB-dependent IL-10 production and restore macrophage homeostasis, ultimately relieving colitis symptoms (Kim et al., 2023).

#### 3.2.3 Lipooligosaccharide

The LOS of *A. muciniphila* differ structurally from the LPS of typical Gram-negative bacteria such as *Escherichia coli*, displaying markedly lower endotoxic activity. Recent studies have revealed that this distinctive LOS architecture engages TLR2 rather than TLR4, leading to enhanced hepatic IL-10 expression and representing a key mechanism by which *A. muciniphila* converts proinflammatory cues into anti-inflammatory signals within the local microenvironment (Garcia-Vello et al., 2024). Although LOS is generally regarded as having low immunogenicity and primarily exerts its regulatory effects through TLR2 rather than TLR4 signaling, evidence suggests that the host TLR4 status can also indirectly influence immune modulation. Liu et al. reported that TLR4 participates in the regulation of *A. muciniphila*-associated immune responses by affecting bacterial colonization and abundance, thereby shaping intestinal immune homeostasis (Liu et al., 2022).

#### 3.2.4 Outer membrane vesicles

In addition, outer membrane vesicles (OMVs), the principal secretory products of *A. muciniphila*, play an important role in modulating host immune responses and attenuating inflammation (Kang et al., 2013; Dao et al., 2016; Wang et al., 2021). These lipid bilayer structures function as delivery vehicles for various immunoregulatory molecules, transporting outer membrane proteins, LOS and other bioactive components directly to intestinal epithelial cells and immune cells, thereby mediating host-microbe interactions. OMVs also decrease intestinal permeability by activating AMP-activated protein kinase (AMPK), which upregulates tight junction (TJ) proteins, suppresses TLR4 and interferon-α (IFN-α) expression, and promotes TLR2 signaling as well as interleukin-4 (IL-4) secretion in Caco-2 cell cultures (Ashrafian et al., 2019; Chelakkot et al., 2018).

Moreover, phospholipid constituents of *A. muciniphila* can be delivered to immune cells via OMVs, facilitating macrophages polarization from pro-inflammatory M1 to the anti-inflammatory M2 phenotype, thus alleviating inflammation and supporting immune homeostasis (Zhu and Deng, 2025). In addition, OMVs further contribute to immune regulation by modulating microRNA expression profiles in dendritic cells. *A. muciniphila* downregulates pro-inflammatory miRNAs such as miR-155 and miR-146a while upregulating anti-inflammatory miRNAs including let-7i, ultimately balancing inflammatory and regulatory pathways (Mofrad et al., 2024).

The immunoregulation induced by OMVs is not a single molecule effect, but the result of multi-component synergy. By preferentially activating TLR2, OMVs elicit a mild and controlled innate immune response that helps restrain excessive inflammation. This property highlights their potential as promising candidates for the development of microbe-derived immunoregulatory therapeutics.

In addition to direct immune modulation, *A. muciniphila* can influence host inflammatory responses through metabolite-mediated mechanisms. The metabolites generated or regulated by *A. muciniphila* can further shape immune signaling and inflammatory cascades, thereby contributing to its overall anti-inflammatory effects.

### 3.3 Metabolite-mediated anti-inflammatory effects

By modulating gut microbial composition and host metabolic pathways, *A. muciniphila* influences the production of numerous inflammation-associated metabolites, thus exerting anti-inflammatory effects (**Figure 2**). Notably, it produces SCFAs such as acetate and propionate, which interact with intestinal epithelial receptors to induce IL-10 secretion and consequently alleviate intestinal inflammation (Zhang et al., 2024a). Additionally, *A. muciniphila* modulates tryptophan metabolism to generate indole derivatives, such as indole-3-acetic acid (IAA) and indole-3-propionic acid (IPA), that activate the aryl hydrocarbon receptor (AhR). AhR activation stimulates interleukin-22 (IL-22) release, enhancing mucosal immune balance, facilitating epithelial regeneration, and suppressing inflammation responses (Shi et al., 2021). Furthermore, specific tripeptides (e.g., Arg-Lys-His, RKH) synthesized by this microorganism have been shown to bind TLR4, thereby inhibiting its activation and mitigating systemic inflammation (Cani et al., 2024).

Additionally, studies have shown that pasteurized *A. muciniphila* strengthens intestinal barrier integrity by lowering serum concentrations of LPS, diamine oxidase (DAO) and D-lactic acid (D-LA). It also modulates inflammation-associated metabolites such as lipids and lipid-like molecules, particularly lysophosphatidylcholines (LysoPCs), while enhancing superoxide dismutase (SOD) activity in colonic tissue, thereby alleviating dextran sulfate sodium (DSS)-induced intestinal oxidative stress (Xue et al., 2023). Moreover, *A. muciniphila* shows a negative correlation with plasma levels of trimethylamine (TMA) and trimethylamine-N-oxide (TMAO), suggesting that by modulating gut microbial metabolites and reducing TMAO production, it may exert anti-atherosclerotic effects (Chen et al., 2016). Further evidence indicates that *A. muciniphila* influences lipid biosynthesis and hepatic inflammation-related gene expression by regulating host metabolic pathways, prevent hepatic steatosis, and alleviates depressive-like behaviors in murine models by regulating the intestinal-brain axis and serotonin (5-HT) levels (Kim et al., 2020; Guo et al., 2024a). At the same time, it can affect the regulation of appetite hormones such as GLP-1, reduce food intake and increase energy consumption, thus improving obesity and related metabolic disorders (Rodrigues et al., 2022; Xu et al., 2020). These metabolites may also reshape the gut microbial ecosystem, thereby contributing to anti-inflammatory effects.

### 3.4 Regulating microbial interactions

*A. muciniphila* exerts therapeutic effects on chronic inflammatory diseases by modulating the composition and diversity of the gut microbiota, thereby optimizing microbial community structure. Several studies have demonstrated that administration of high doses of *A. muciniphila* can effectively reshape intestinal microbial profiles. Analysis of fecal 16S rRNA sequences from treated mice revealed an increased relative abundance of the phylum *Firmicutes*, including members of the families *Lachnospiraceae* and *Ruminococcaceae*, accompanied by a decline in *Bacteroidetes*, thereby contributing to the alleviation of inflammation (Wu et al., 2017b). Likewise, Xue et al. found that supplementation with either live or pasteurized *A. muciniphila* elevated the proportions of *Bacteroidetes*, *Verrucomicrobia*, *Firmicutes* and *Actinobacteria*, while decreasing *Proteobacteria* at the phylum level (Xue et al., 2023).

Pasteurized *A. muciniphila* has also been shown to alleviate alcohol-induced gut dysbiosis, leading to higher relative abundances of *Bacteroides*, *Akkermansia*, *Escherichia* and *Parabacteroides* at the genus level (Cheng et al., 2024). Moreover, by degrading mucin and producing metabolites such as SCFAs, *A. muciniphila* can modulate populations of SCFA-dependent microorganisms (Belzer and De Vos, 2012). At the family level, its supplementation increases *Akkermansiaceae*, *Muribaculaceae*, *Bacteroidaceae* and *Bifidobacteriaceae*, while decreasing *Moraxellaceae* and *Pseudomonadaceae* (Xue et al., 2023).

Overall, the presence of *A. muciniphila* supports gut ecological stability by suppressing inflammation-associated pathogenic taxa. Since the overgrowth of such bacteria is commonly linked to chronic inflammation and metabolic dysfunction, restoring microbial homeostasis through *A. muciniphila* supplementation may help alleviate symptoms of these disorders (Ioannou et al., 2025).

Overall, *A. muciniphila* strengthens the intestinal barrier, thereby limiting excessive immune activation and creating a microenvironment that favors microbial stability. Meanwhile, *A. muciniphila*–associated metabolites can directly modulate immune signaling pathways and inflammatory cascades, contributing to immune regulation. These metabolites may also influence the composition and function of the gut microbiota, promoting microbial diversity and ecological balance. In turn, a more stable and diverse microbial community further supports metabolite production and reinforces barrier integrity, forming a dynamic feedback network. The coordinated interaction among these mechanisms ultimately enables *A. muciniphila* to exert anti-inflammatory effects. Although numerous studies have highlighted the beneficial effects of *A. muciniphila* in inflammatory disorders, emerging evidence indicates that under certain host genetic backgrounds, disease states, or microbial ecological conditions, the bacterium may also exert pro-inflammatory effects.

### 3.5 Dual roles of *A. muciniphila* in inflammation

Emerging evidence suggests that host immune status, genetic susceptibility, and the infectious environment may all influence the effects of *A. muciniphila* on the host. For instance, In IL-10-deficient mice, *A. muciniphila* induced colitis and facilitated bacterial translocation (Seregin et al., 2017). In TAGAP-deficient mice, an increased abundance of *A. muciniphila* has been reported to exacerbate DSS-induced colitis (He et al., 2023). In *Apc* transgenic mouse models of colorectal cancer (CRC), supplementation with *A. muciniphila* significantly increased tumor burden, indicating a possible role in promoting intestinal inflammation and tumor progression (Dingemanse et al., 2015). In Cyp27b1-deficient mice, the genetic background associated with vitamin D deficiency alters intestinal immune homeostasis, leading *A. muciniphila* to exhibit pro-inflammatory properties (Zhu et al., 2019). In mouse models of *Salmonella* infection, *A. muciniphila* exacerbated intestinal inflammation (Chiantera et al., 2023).

In addition to host factors, specific disease background can also influence the functional role of *A. muciniphila*. In genetic CRC models, *A. muciniphila* exhibits anti-tumor effects, whereas in the inflammation-associated CRC AOM/DSS model, it aggravates inflammation and promotes tumor development (Soheilipour et al., 2025). During the microbiota reconstitution phase following antibiotic-induced dysbiosis, the overgrowth of *A. muciniphila* can exert pro-inflammatory effects and aggravate antibiotic-associated intestinal inflammation, and this effect disappears as the microbiota recovers (Wang et al., 2022; Qu et al., 2023). A recent study also found that *A. muciniphila* exacerbates inflammatory responses in hosts with radiation-induced intestinal injury (Wang et al., 2025a).

Beyond host factors and disease contexts, the biological effects of *A. muciniphila* are also influenced by its interactions with other gut microorganisms. Studies have suggested that *A. muciniphila* interacts with sulfate-reducing bacteria to form a synergistic pro-inflammatory network, which aggravates intestinal inflammation and contributes to the pathogenesis of PD (Hertel et al., 2019; Romano et al., 2021; Murros, 2022). In patients with PD, dysbiosis characterized by the overgrowth of *A. muciniphila* and a reduction in butyrate-producing bacteria promotes the development of inflammation (Perez-Pardo et al., 2019). Similarly, mouse models have shown that *A. muciniphila* can interact with butyrate-producing bacteria to promote intestinal inflammation and contribute to the pathogenesis of PD (Dodiya et al., 2020).

Notably, treatment with metformin has been consistently reported to increase the abundance of *A. muciniphila* in both human and animal studies (Shin et al., 2014; Wu et al., 2017a; Ke et al., 2021). During metformin treatment, the increase in *A. muciniphila* abundance is considered part of an overall microbiota restructuring rather than the excessive expansion of a single species, thereby exerting a positive regulatory effect. Current evidence suggests that metformin-induced enrichment of *A. muciniphila* is generally regarded as beneficial to host health, and no clear adverse effects have been identified. Nevertheless, a genome-scale metabolic modeling study proposed a distinct hypothesis. Through computational simulations, Rosario et al. suggested that dysbiosis resulting from metformin-induced overexpansion of *A. muciniphila* may potentially have unfavorable metabolic consequences for the host (Rosario et al., 2018).

Taken together, although *A. muciniphila* is widely regarded as a beneficial bacterium and its enrichment has often been associated with positive metabolic effects, there are also studies suggesting that its effects are highly context-dependent and may shift toward pro-inflammatory outcomes under specific host, disease, microbial, or pharmacological conditions. Therefore, future research should consider the various factors mentioned above when evaluating the therapeutic potential of *A. muciniphila*.

## 4 Applications related to *A. muciniphila*

### 4.1 The therapeutic value of *A. muciniphila* against inflammatory diseases

**Figure 3:**
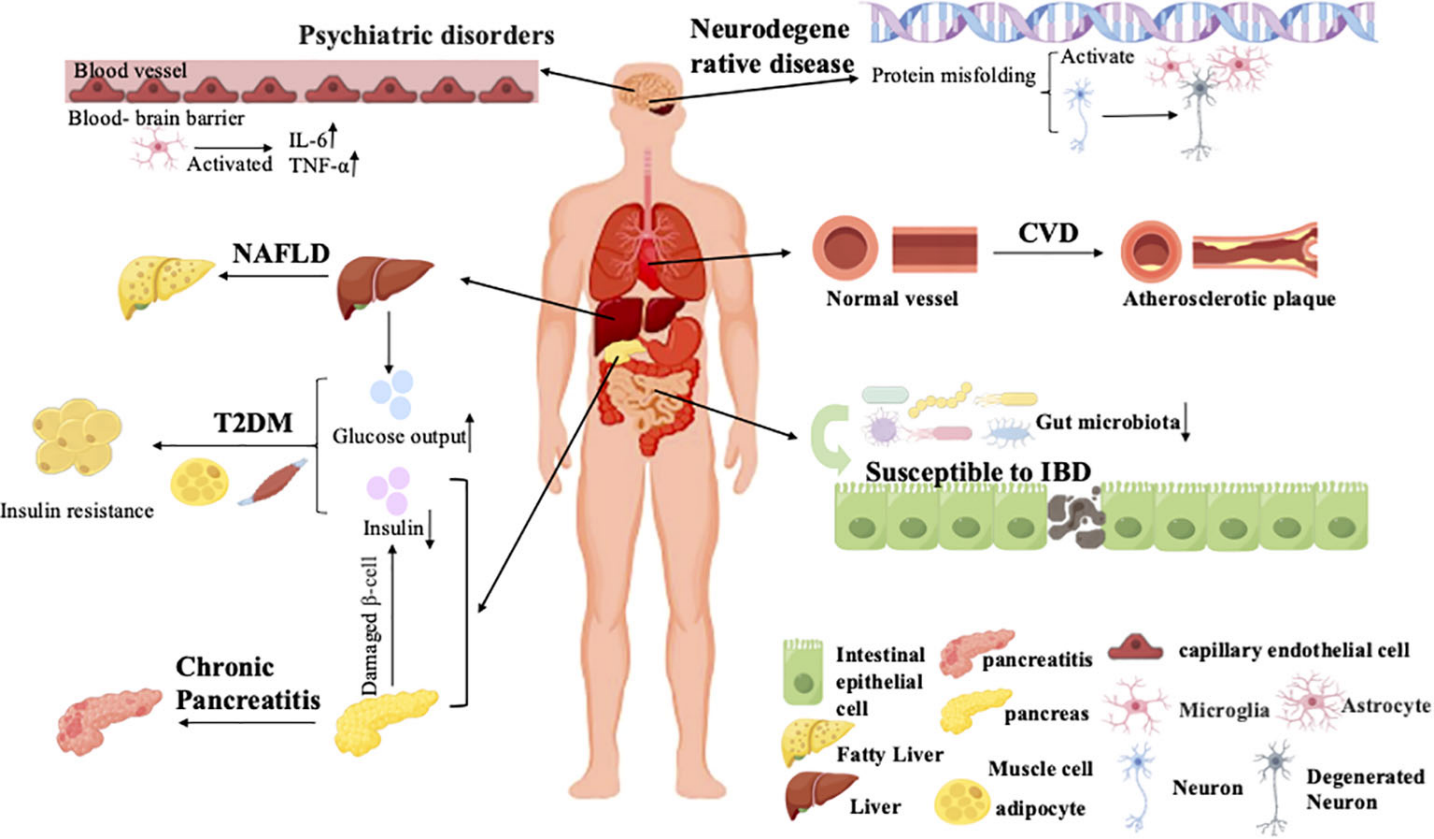
Chronic inflammation-associated diseases. Inflammatory bowel disease (IBD) is widely recognized to involve intestinal barrier dysfunction and immune dysregulation. Nonalcoholic steatohepatitis (NASH) represents the active inflammatory stage of nonalcoholic fatty liver disease (NAFLD). Chronic pancreatitis is characterized by persistent pancreatic inflammation, fibrosis, and impaired exocrine activity. In type 2 diabetes mellitus (T2DM), insulin resistance is closely linked to metabolic inflammation. Neurodegenerative disorders arise from protein misfolding and aggregation, which activate microglia and astrocytes. Patients with chronic depression exhibit elevated inflammatory markers in both the peripheral and central nervous systems.

*A. muciniphila* is closely associated with the onset and progression of various inflammatory disorders. Alterations in its abundance can disrupt intestinal barrier integrity, impair immune equilibrium, and influence systemic inflammatory status, thereby contributing to the pathogenesis of conditions such as mental and neurodegenerative diseases, T2DM, chronic pancreatitis, Non-alcoholic fatty liver disease (NAFLD), and IBD through a multi-organ axis (**Figure 3**).

In patients with inflammatory diseases, the abundance of *A. muciniphila* is typically lower than that in healthy subjects, suggesting a potential protective role in suppressing inflammation during disease progression. Its depletion may compromise intestinal barrier integrity, allowing translocation of pathogens and toxic molecules across the epithelium into the circulation, thereby promoting systemic disorders (Zhang et al., 2021a). Evidence from both animal experiments and clinical studies demonstrates that restoring *A. muciniphila* levels through dietary interventions, prebiotics, or probiotic supplementation improves metabolic health, decreases inflammatory biomarkers, and can partially prevent or ameliorate inflammation-associated diseases (Dao et al., 2016; Guo et al., 2024b). Owing to its ability to modulate gut microbial balance, reinforce barrier function, and suppress inflammatory signaling with minimal adverse effects, *A. muciniphila* represents a promising and safe therapeutic candidate for chronic inflammation–related disorders (**Table 1**).

**Table 1:** Therapeutic effects of *A. muciniphila* on chronic inflammation-related diseases.

Study type	Disease	Model/subjects	Intervention	Dose/concentration	Duration	Main findings	Reference
*In vitro* studies	Inflammatory bowel disease	Caco-2 cells(LPS)	*A. muciniphila*	50μg/mL	12 hours	TNF-α, IL-1β, IL-6↓. ZO-1, Occludin↑	(Zhao et al., 2025)
		Caco-2 cells(LPS)	*A. muciniphila*	1×10^7^ CFU/mL	12 hours	TGF-β, IL-10, ZO-1, Occludin↑. TNF-α, IL-1β, IL-6, IL-8↓	(Shi et al., 2022)
		HT-29 cells(TNF-α)	*A. muciniphila* ATCC BAA-835	–	8 hours	IL-8↓	(Zhai et al., 2019b)
		IPEC-J2 cells(TNF-α)	Live and heat-killed *A. muciniphila*	10^8–^10^9^ copies/mL	7.5 hours	TNF-α, IL-1β, IL-6, IL-8↓. ZO-1, Occludin↑	(Luo et al., 2021)
	Neurodegenerative diseases	EGC CRL-2690 human enteric glial cells(RS09)	*A. muciniphila*	5×10^6^ CFU/mL	6 hours	GDNF, NEDD4↑. CX43↓	(Mu et al., 2025)
		BV2 microglial cells(LPS)	*A. muciniphila* outer membrane vesicles	200μg/mL	24 hours	MMP-9↓	(Gao et al., 2024)
		HT22 mouse hippocampal neuronal cells	*A. muciniphila* metabolites	5×10–^6^ mol/mL	26 hours	DRP1↓	(Wang et al., 2025b)
	Obesity and diabetes	3T3-L1 adipocytes	Pasteurized *A. muciniphila*	1×10^8^ CFU/mL	6 days	PPARγ, C/EBPα, CD36↓	(Yang et al., 2020)
		3T3-L1 adipocytes	*A. muciniphila* cell lysate	1×10^7^ CFU/mL	6 days	SERPINA3G↑	(Lee et al., 2022)
		NCI-H716 L cells	P9 protein secreted by *A. muciniphila*	500μg/mL	60 min	GLP-1↑	(Arukha et al., 2025)
	NAFLD	LX-2 hepatic stellate cells(TGF-β1)	*A. muciniphila*	–	24 hours	SLC40A1, COL1A1↓	(Ahmadi Badi et al., 2025)
		LX-2 hepatic stellate cells(LPS)	*A. muciniphila* outer membrane vesicles	50μg/mL	24 hours	tlr-5, tlr-9↓	(Raftar et al., 2022)
		Colon organoid–gut microbiota co-culture system	*A. muciniphila*	1×10^6^ CFU/mL	4–6 days	α-Ketoisovaleric acid↑	(Liu et al., 2025)
Animal studies	Inflammatory bowel disease	C57BL/6N mouse(DSS/CRS)	Oral gavage	1×10^8^ CFU/mL	14 days	Supplementation of *A. muciniphila* can ameliorate depressive-like symptoms and reduce colitis in mice.	(Chen et al., 2021)
		C57BL/6 female mouse (DSS)	Oral gavage	1×10^10^ CFU/mL	5 days	AKK@GPMGs: *Lactobacillaceae*↑ *Muribaculaceae*↑ *Akkermansiaceae*↑ *Enterobacteriaceae*↓ *Desulfovibrionaceae*↓	(Zhang et al., 2024b)
		C57BL/6J male mouse(DSS)	Oral gavage	5×10^7^-2.5×10^8^ CFU/mL	3 days	AKK@MFe_3_O_4_: *L. murinus*↑ IL-1β↓ TNF-α↓ IL-10↑ IL-4↑	(Wang et al., 2023a)
	Neurodegenerative diseases	APP/PS1 mouse(HFD)	Oral gavage	5×10^9^ CFU/mL	6 months	Improvement of cognitive function in mice.	(Ou et al., 2020)
		5×FAD female mouse	Oral gavage	2×10^8^-4×10^8^ CFU/mL	8 weeks	Improve cognitive impairment and reduce blood-brain barrier permeability	(Wang et al., 2025b)
	Obesity and diabetes	C57BL/6J male mouse(HFD)	Oral gavage	10^8–^10^9^ CFU/mL	4 weeks	Supplementation of *A. muciniphila* improves metabolic status in obese and diabetic mice.	(Rodrigues et al., 2022)
		C57BL/6J male mouse(HFD)	Oral gavage	2×10^8^ CFU/mL	4 weeks	Supplementation of *A. muciniphila* alleviates HFD-Induced obesity in mice.	(Everard et al., 2013)
		C57BL/6 male mouse(HFD)	Oral gavage	10μg/mouse	2 weeks	Supplementation of *A. muciniphila* improves metabolic status in obese and diabetic mice.	(Chelakkot et al., 2018)
	NAFLD	C57BL/6N male mouse(HFD)	Oral gavage	10^8–^10^9^ CFU/mL	10 weeks	*A. muciniphila* can prevent the development of NAFLD.	(Kim et al., 2020)
	Psychiatric disorders	C57BL/6J male mouse(alcohol, CUMS)	Oral gavage	2.5×10^9^ CFU/mL	2–5 weeks	Supplementation of *A. muciniphila* can ameliorate depressive-like symptoms in mice.	(Guo et al., 2024a)
		C57BL/6 male mouse(CRS)	Oral gavage	5 × 10^8^ CFU/mL	3 weeks	Supplementation of *A. muciniphila* can ameliorate depressive-like symptoms in mice.	(Ding et al., 2021)
		C57BL/6 male mouse(antibiotics)	Oral gavage	7.5 × 10^9^ CFU/mL	2 weeks	Supplementation of *A. muciniphila* can improve anxiety and depressive-like symptoms in mice.	(Sun et al., 2023)
		Male offspring of Wistar rats(VPA)	Oral gavage	4 × 10^9^ CFU/mL	30 days	Supplementation of *A. muciniphila* can improve autism like behavior and neuroinflammation	(Adıgüzel et al., 2026)
		BTBR mouse	Oral gavage	1 × 10^10^ CFU/mL	4 weeks	Supplementation of *A. muciniphila* alone did not improve autism like behavior	(Yang et al., 2025)
Clinical studies	Inflammatory bowel disease	20 UC, 26 CD, 20 CON	–	–	–	In IBD: *A. muciniphila*↓ *R. gnavus*↑ *R. torques*↑	(Png et al., 2010)
		23 UC, 31 CD, 17 CON	–	–	–	In CD: *F. prausnitzii*↓ *A. muciniphila*↓	(Lopez-Siles et al., 2018)
		78 UC, 17 CD, 96 CON	–	–	–	In CD: *A. muciniphila*↑ In UC: *A. muciniphila*↓	(Danilova et al., 2019)
		14 QUC, 20 AC, 20 HC	–	–	–	In AC: *A. muciniphila*↓	(Earley et al., 2019)
		105 UC patients in remission	–	–	–	The abundance of *A. muciniphila* and *P. distason* is correlated with shorter remission periods	(Mendes-Frias et al., 2024)
		32 UC, 31 HC	–	–	–	In UC: *A. muciniphila*↓	(Bajer et al., 2017)
		43 UC, 41 CD, 42 HC	–	–	–	In CD: *A. muciniphila*↓ In UC: *A. muciniphila*↓	(Lo Sasso et al., 2021)
		30 IBD, 41 HC	–	–	–	In IBD: *A. muciniphila*↓	(Presti et al., 2019)
		20 UC, 26 CD, 20 HC	–	–	–	In CD: *A. muciniphila*↓ In UC: *A. muciniphila*↓	(Png et al., 2010)
	Obesity and diabetes	11 overweight, 38 obese	calorie restriction	–	–	In obese people, those with high baseline *A. muciniphila* abundance are more metabolic.	(Dao et al., 2016)
		11 placebo group, 12 pasteurized bacteria group, 9 live bacteria group	oral powder	10^10^ CFU/mL	3 months	In pasteurized bacteria group: insulin sensitivity has improved significantly.	(Depommier et al., 2019)
		29 placebo group, 29 AKK-WST01 group	oral powder	1-5×10^10^ CFU/mL	12 weeks	The clinical improvement was significant in the low baseline group.	(Zhang et al., 2025)
		181 T2MD, 187 HC	–	–	–	In T2MD: *F. prausnitzii*, *E. rectale*↓	(Qin et al., 2012)
		53 T2MD, 49 IGT, 43 NGT	–	–	–	In T2MD: Clostridiales↓, *Lactobacillus*↑	(Karlsson et al., 2013)
	Psychiatric disorders	23 ASD, 22 SIB, 9 CON	–	–	–	In ASD: *A. muciniphila*↓	(Wang et al., 2011)
		112 children	–	–	–	In *A. muciniphila* detection group: the depression score↓	(Varesi et al., 2023)

UC, Ulcerative colitis; CD, Crohn’s disease; CON, Unrelated community controls; HC, healthy controls; QUC, quiescent UC; AC, active UC; IGT, impaired glucose tolerance; NGT, normal glucose tolerance; DSS, Dextran sulfate sodium; CRS, Chronic Restraint Stress; ASD, Autism spectrum disorder; SIB, Typically developing siblings; RS09, TLR4 agonist; VPA, valproic acid.

#### 4.1.1 Inflammatory bowel disease

IBD is characterized by chronic intestinal inflammation and disruption of the mucosal barrier. Its occurrence and development are closely associated with gut microbiota imbalance and abnormal immune activation (Flynn and Eisenstein, 2019). Increasing evidence indicates that *A. muciniphila* reduces both intestinal and systemic inflammation by reinforcing intestinal barrier integrity and limiting the generation of inflammatory mediators (Zhang et al., 2021b; Zheng et al., 2023a).

*In vitro* studies have demonstrated that *A. muciniphila* significantly reduces the expression of pro-inflammatory cytokines associated with IBD, including TNF-α, IL-1β, and IL-6, in LPS-induced Caco-2 cells by inhibiting the MAPK signaling pathway. Moreover, it restores the expression of tight junction proteins ZO-1 and Occludin, thereby improving intestinal barrier function (Shi et al., 2022; Zhao et al., 2025). *In vitro* experiments by Zhai et al. demonstrated that *A. muciniphila* ATCC BAA-835 significantly inhibits IL-8 production in TNF-α–stimulated HT-29 cells (Zhai et al., 2019b). Similarly, Zhang et al. reported that both live and heat-killed *A. muciniphila* significantly reduce the expression of pro-inflammatory cytokines and upregulate tight junction proteins in a TNF-α–induced inflammatory model of porcine intestinal epithelial cells (IPEC-J2) (Luo et al., 2021).

In animal experimental models, administration with *A. muciniphila* has been shown to restore its reduced abundance in C57BL/6 mice with DSS-induced chronic colitis, while promoting gut microbial homeostasis and enhancing the integrity of the intestinal epithelial barrier, thereby alleviating chronic inflammation (Chen et al., 2021). Beyond direct oral supplementation, targeted delivery systems have been developed to facilitate its localization at inflamed sites and enhance colonization efficiency, thereby improving therapeutic efficacy (Wang et al., 2023a; Zhang et al., 2024b).

Several human cohort studies have investigated the association between *A. muciniphila* and IBD. For example, Earley et al. reported that the abundance of *A. muciniphila* was significantly reduced in patients with active ulcerative colitis (Earley et al., 2019). Moreover, Mendes-Frias et al. recruited 105 patients with ulcerative colitis in remission and demonstrated that the levels of *A. muciniphila* were associated with the remission state and may potentially predict disease relapse (Mendes-Frias et al., 2024). Other clinical microbiome studies have reported that the abundance of *A. muciniphila* is reduced in patients with IBD compared with healthy individuals (Png et al., 2010; Bajer et al., 2017; Presti et al., 2019; Lo Sasso et al., 2021). However, one study reported an opposite finding, showing that *A. muciniphila* abundance was decreased in patients with ulcerative colitis (UC) but increased in those with Crohn’s disease (CD) (Danilova et al., 2019).

However, most of these studies are observational and based on microbiome analyses rather than direct probiotic intervention trials. Therefore, although current clinical data suggest a potential association between *A. muciniphila* and intestinal inflammation, clinical trials evaluating the therapeutic effects of *A. muciniphila* in patients with IBD remain limited.

#### 4.1.2 Obesity and T2DM

Obesity and T2DM are metabolic disorders characterized by dysregulated energy homeostasis, chronic low-grade inflammation and insulin resistance. Their occurrence and development are closely associated with alterations in gut microbial composition (Himanshu et al., 2020). *A. muciniphila* contributes to the regulation of host energy metabolism by influencing fatty acid storage and utilization, thereby exerting beneficial effects on metabolic disturbances such as obesity and diabetes (Rodrigues et al., 2022; Xu et al., 2020).

*In vitro* studies have demonstrated that pasteurized *A. muciniphila* markedly inhibits lipid droplet accumulation in 3T3-L1 adipocytes and downregulates lipogenic markers and lipid-synthesizing enzyme genes (Yang et al., 2020). Using multi-omics analyses, Song et al. showed that *A. muciniphila* cell lysates reduce the expression of proteins involved in adipocyte differentiation, fatty acid metabolism, and energy metabolism in 3T3-L1 cells (Lee et al., 2022). Furthermore, in NCI-H716 L cells, P9 improves glucose homeostasis and body weight control by activating the GLP-1 receptor pathway (Arukha et al., 2025).

In animal models, daily intragastric administration of 10^8–^10^9^ CFU/mL of live *A. muciniphila* improved metabolic profiles in obese and diabetic mice, whereas oral delivery of 2×10^8^ bacterial cells per day alleviated high fat diet (HFD)-induced obesity (Everard et al., 2013; Rodrigues et al., 2022). Administration of OMVs also improved glucose tolerance and mitigated weight gain in HFD-induced diabetic mice (Chelakkot et al., 2018).

Clinical studies have shown that dietary intervention improves metabolic parameters in overweight or obese individuals through modulation of the gut microbiota (Dao et al., 2016). Depommier et al. documented that daily oral administration of 10^10^ CFU of *A. muciniphila* is safe in overweight and obese human volunteers (Depommier et al., 2019). Zhang et al. further reported that the ameliorative impact of *A. muciniphila* (AKK-WST01) supplements on weight and metabolic indicators in overweight or obese patients with T2DM were significant only among those with low baseline intestinal abundance of the strain, while no improvement was observed in individuals with higher baseline levels (Zhang et al., 2025). Multiple clinical observational studies have found that *A. muciniphila* abundance is negatively associated with obesity and insulin resistance (Qin et al., 2012; Karlsson et al., 2013).

#### 4.1.3 Nonalcoholic fatty liver disease

NAFLD is characterized by excessive lipid accumulation in the liver, accompanied by chronic low-grade inflammation and metabolic disorders. Its more severe manifestation is NASH (Seko et al., 2018). Studies have demonstrated that *A. muciniphila* can modulate L-aspartate metabolism via the gut-liver axis, thereby ameliorating hepatic steatosis associated with metabolic disturbances (Rao et al., 2021).

*In vitro* studies demonstrated that *A. muciniphila* and its OMVs markedly suppress TLR-2 and TLR-4 gene expression, inhibit activation of hepatic stellate cells, and alleviate liver fibrosis in LPS-stimulated human LX-2 cells (Raftar et al., 2022; Ahmadi Badi et al., 2025). Li et al. established an *in vitro* co-culture system of colonic organoids and gut microbiota, revealing that *A. muciniphila* ameliorates hepatic lipid metabolism via the gut-liver axis (Liu et al., 2025).

In animal models, Kim et al. reported that gavage C57BL/6N mice with 10^8–^10^9^ CFU/mL of *A. muciniphila* could prevent fatty liver disease (Kim et al., 2020). Similarly, Rao et al. showed that *A. muciniphila* supplementation effectively reversed hepatic steatosis in obese mice subjected to a high-fat, high-cholesterol (HFC) diet (Rao et al., 2021).

To date, clinical trial data directly assessing *A. muciniphila* in patients with NAFLD are still limited. Most evidence comes from animal model studies.

#### 4.1.4 Neurodegenerative and psychiatric disorders

In recent years, the gut-brain axis has been regarded as an important channel connecting intestinal microecology with the function of the central nervous system. Chronic inflammation plays a pivotal role in the occurrence and development of a variety of neurodegenerative (Beurel et al., 2020; Ciurea et al., 2023). However, clinical trials on *A. muciniphila* in neurodegenerative diseases are currently very limited, with most studies focusing primarily on *in vitro* research and animal experiments.

*In vitro* studies demonstrated that in the human enteric glial cell line EGC CRL-2690, *A. muciniphila* mitigates PD-related enteric glial dysfunction by modulating GDNF signaling and upregulating NEDD4 to promote CX43 ubiquitination and degradation (Mu et al., 2025). Gao et al. further showed that in LPS-induced BV2 microglial cells, treatment with OMVs inhibits microglial activation and inflammatory cytokine release via the TLR2/4 pathway (Gao et al., 2024). Wang et al. reported that *A. muciniphila* metabolites enhance PINK1/Parkin-mediated mitophagy in HT22 mouse hippocampal neurons through GPR43, thereby ameliorating mitochondrial dysfunction in Alzheimer’s disease (Wang et al., 2025b).

In murine models of Alzheimer’s disease, *A. muciniphila* administration reduces β-amyloid accumulation, ameliorates glucose intolerance and epithelial injury, and partially ameliorate cognitive impairment (Ou et al., 2020).

In terms of psychological disorders, emerging evidence suggested that *A. muciniphila* may influence mood regulation and behavior through the gut-brain axis (Cheng et al., 2021).

In animal models, intervention with *A. muciniphila* has been shown to significantly improve depressive-like behaviors and restore neuroendocrine function in mice (Ding et al., 2021; Sun et al., 2023). Other studies reported that *A. muciniphila* can ameliorate stereotyped behaviors, social deficits, and memory impairments in rat models of autism (Adıgüzel et al., 2026). However, in BTBR mice, Yang et al. observed an opposite effect, where *A. muciniphila* intervention partially counteracted the social improvement induced by *Lactobacillus rhamnosus* GR-1 (Yang et al., 2025). Guo et al. documented that oral gavage of *A. muciniphila* (2.5×10^9^ CFU/200 μL) in C57BL/6J SPF mice ameliorated depressive-like behaviors induced by chronic alcohol exposure and chronic unpredictable mild stress (CUMS) (Guo et al., 2024a).

Comparative analyses of gut microbiota in children with autism spectrum disorder (ASD) and neurotypical controls have revealed a markedly lower abundance of *A. muciniphila* in individuals with ASD and their siblings (Wang et al., 2011). A birth cohort study showed that the presence of *A. muciniphila* attenuated the association between specific prenatal metal exposures and depressive symptoms in children (Varesi et al., 2023). Currently, research on the application of *A. muciniphila* in psychiatric disorders is very limited, consisting predominantly of observational studies, while interventional trials are still in the exploratory stage.

Overall, current evidence from experimental models and human studies supports a beneficial role of *A. muciniphila* in inflammatory diseases. However, differences in experimental design, host metabolic status, diet, microbial strains, and intervention duration may influence study outcomes. Future research should aim to standardize experimental conditions and conduct large-scale clinical trials to better clarify the therapeutic potential of *A. muciniphila*.

### 4.2 Challenges for clinical translation

#### 4.2.1 Safety considerations

Although *A. muciniphila* is widely considered a promising next-generation probiotic and early clinical studies have suggested favorable safety profiles, several safety-related issues should be carefully considered before its large-scale clinical application.

One important concern is that the effects of *A. muciniphila* may be highly host-dependent. While most studies report beneficial metabolic and anti-inflammatory effects, evidence from certain experimental models suggests that *A. muciniphila* may exacerbate inflammation under specific host conditions, such as immune deficiencies or disrupted gut environments. These findings indicate that the safety of *A. muciniphila* supplementation may vary depending on host immune status, disease background, and microbial ecosystem.

In addition, strain-specific differences may influence both efficacy and safety. Different strains of *A. muciniphila* may possess distinct metabolic capacities, immunomodulatory properties, and host interactions (Guo et al., 2017; Ouwerkerk et al., 2022). For example, *A. muciniphila* FSDLZ36M5 exhibited a pronounced protective effect in a mouse model of ulcerative colitis, whereas *A. muciniphila* FSDLZ20M4 showed a tendency to exacerbate inflammation (Liu et al., 2021). The anti-inflammatory effect of *A. muciniphila* ATCC BAA-835 was superior to that of *A. muciniphila* 139 in a mouse model of chronic colitis, whereas its ameliorative effect in a model of metabolic inflammation was inferior to that of a newly isolated *Akkermansia* sp. DSM 33459 (Ring et al., 2019; Zhai et al., 2019b; Kumar et al., 2022). Therefore, careful evaluation of strain-specific characteristics is necessary to ensure the safety of candidate strains intended for therapeutic use.

#### 4.2.2 Supplementation strategies for *A. muciniphila*

Both clinical and preclinical studies have verified that administration with either live or pasteurized *A. muciniphila* holds promising potential for improving gut health and alleviating symptoms associated with chronic inflammation (Fang et al., 2021; Mruk-Mazurkiewicz et al., 2024). Current intervention strategies primarily include oral administration of viable cells, pasteurized formulations, and methods that stimulate its proliferation in the gut through prebiotic supplementation or dietary modification (EFSA Panel on Nutrition, Novel Foods and Food Allergens (NDA) et al., 2021; Sorbara and Pamer, 2022; Zhang et al., 2024c). These strategies aim to increase the abundance of *A. muciniphila* within the intestinal microbiota and thereby enhance its therapeutic efficacy against inflammatory diseases.

Live *A. muciniphila* preparations are commonly delivered as oral capsules or lyophilised powders, enabling colonization within the intestinal mucus layer and sustained biological activity. Evidence indicates that these formulations alleviate inflammatory phenotypes associated with inflammatory bowel disease, metabolic syndrome and certain neuropsychiatric disorders (Depommier et al., 2019). However, the practical application of viable strains faces several limitations, including susceptibility to degradation by gastric acid and bile salts, which compromise colonization efficiency, and the potential for excessive immune responses in some individuals, posing safety concerns (Zhai et al., 2019a). In contrast, pasteurized *A. muciniphila* retains key surface components, such as the outer membrane protein Amuc_1100 and LOS, allowing it to interact with host receptors such as TLR2 and activate downstream signaling pathways while avoiding the risks associated with live bacterial colonization (Plovier et al., 2017). Recent preclinical and clinical evidence suggests that pasteurized *A. muciniphila* may even outperform the live form in ameliorating obesity and metabolic dysfunction, as evidenced by greater reductions in body weight and improved glucose tolerance in HFD-induced obese mice (Plovier et al., 2017; Yan et al., 2021).

Although pasteurized bacteria are more stable and safer, the precise mechanisms of action of their bioactive molecules and their clinical translatability remain to be fully elucidated (Derrien et al., 2017; Depommier et al., 2019). To address these challenges, recent research has increasingly focused on the derivatives of *A. muciniphila*, including its OMVs, key structural proteins and metabolic products. These components can partially replace or simulate the functions of the bacteria themselves, avoiding the risks of live bacteria while retaining or even enhancing their immunomodulatory and metabolic improvement effects (Plovier et al., 2017; Chelakkot et al., 2018).

#### 4.2.3 Derivatives of *A. muciniphila*

Compared with whole-bacterium intervention, the derivatives of *A. muciniphila* have advantages such as defined pharmacological targets, stable preparations and clearer safety boundaries, and are becoming promising alternatives for inflammation-related diseases. Both *in vivo* and *in vitro* experiments have revealed that OMVs from *A. muciniphila* are able to carry membrane proteins and LOS, which are taken up by intestinal epithelial cells to directly remodel the barrier and adherens junctions. In DSS-induced colitis models, OMVs administration markedly reduced mucosal inflammation, restored tight junction integrity and corrected microbial dysbiosis, suggesting their potential use as “postbiotic exosomes” for mucosal immune regulation (Chelakkot et al., 2018; Zheng et al., 2023b). In the obesity and metabolic inflammation model, OMVs often perform better than whole bacteria in improving the intestinal barrier, reducing adipose tissue inflammation, and improving metabolic parameters, and can regulate the expression of metabolism-related genes in Caco-2 cells (Ashrafian et al., 2019).

Recent mouse studies further revealed that specific membrane proteins of *A. muciniphila*, such as Amuc_1409, can increase peripheral Treg/IL-10 levels while mitigating pancreatic and systemic inflammation, highlighting the therapeutic potential of bacterial derivatives in pancreatitis (Xie et al., 2025). Increasing evidence also indicates that *A. muciniphila* and its metabolites can alleviate neuroinflammation by reducing peripheral and central inflammation and modulating the SCFA-GPR axis. Corresponding animal models of Parkinson’s disease and Alzheimer’s disease have reported improvements in both cognitive and motor performance following such interventions (Qiao et al., 2024; Wang et al., 2025b).

#### 4.2.4 Regulatory considerations

In addition to safety, administration strategies, and formulation stability, regulatory considerations represent another important factor influencing the clinical translation of *A. muciniphila*. As a member of the emerging class of next-generation probiotics, *A. muciniphila* does not yet fall neatly within traditional regulatory categories for probiotics, dietary supplements, or pharmaceutical biologics. Consequently, the regulatory pathways for the clinical development and commercialization of *A. muciniphila*-based products remain under active discussion.

Another key regulatory challenge involves the establishment of standardized manufacturing and quality control criteria. Furthermore, large-scale, well-designed clinical trials will be necessary to demonstrate the therapeutic efficacy and long-term safety of *A. muciniphila*-based interventions in specific disease populations.

Therefore, the establishment of clear regulatory frameworks and standardized development guidelines will be critical for facilitating the safe and effective clinical translation of *A. muciniphila*.

## 5 Perspectives

Current management of chronic inflammatory disorders still relies primarily on conventional therapies, including anti-inflammatory agents, immunosuppressants, and biologics, whose long-term use is often associated with adverse effects such as drug resistance and organ toxicity (Gómez-García et al., 2019; Ozer et al., 2025). Consequently, there is an urgent need to develop safer and more effective strategies for prevention, diagnosis, and treatment. As a novel probiotic candidate, *A. muciniphila* has demonstrated potent anti-inflammatory and protective activities across multiple chronic inflammatory conditions. It maintains intestinal homeostasis and inhibits the occurrence and development of chronic inflammation through multiple synergistic mechanisms. The interplay among these mechanisms not only demonstrates significant therapeutic potential in systemic inflammatory diseases but also provides novel perspectives into immune regulation within the central nervous system and neuropsychiatric disorders.

This review systematically integrates current understanding of the role of *A. muciniphila* in chronic inflammatory diseases and examines its anti-inflammatory properties across multiple dimensions, spanning molecular mechanisms to translational applications. Based on existing studies, we delineate how *A. muciniphila* exerts anti-inflammatory effects through interconnected pathways, including intestinal barrier protection, immunomodulation, metabolite-mediated signaling, and microbial community remodeling. These mechanisms are correlated with experimental and clinical evidence in diverse inflammation-associated disorders, thereby establishing a coherent framework linking *A. muciniphila* to disease modulation. Beyond its potential as live bacteria, this review also highlights recent advances in pasteurized formulations and bioactive derivatives, discussing the distinct advantages of different delivery formats. By integrating mechanistic insights with translational perspectives, this work aims to provide a systematic and forward-looking assessment of the unique biological value of *A. muciniphila* in chronic inflammatory diseases.

In conclusion, *A. muciniphila*, as a new generation of potential probiotics, exhibits multidimensional regulatory roles in inflammatory diseases. Future research, propelled by multi-omics and synthetic biology, will expand from natural strain screening to targeted probiotic interventions and engineered strain development, enabling tailored therapies for specific disease states or host phenotypes. Combination therapies are anticipated to synergize with other modalities, offering novel multi-target approaches for complex chronic inflammation. As derivative applications (e.g., pasteurized formulations, bioactive metabolites) are explored, *A. muciniphila*is poised to bridge basic discovery and clinical practice, pioneering innovative avenues for inflammatory intervention.
